# Extra-Nuclear Signalling of Estrogen Receptor to Breast Cancer Cytoskeletal Remodelling, Migration and Invasion

**DOI:** 10.1371/journal.pone.0002238

**Published:** 2008-05-21

**Authors:** Maria Silvia Giretti, Xiao-Dong Fu, Giovanni De Rosa, Ivana Sarotto, Chiara Baldacci, Silvia Garibaldi, Paolo Mannella, Nicoletta Biglia, Piero Sismondi, Andrea Riccardo Genazzani, Tommaso Simoncini

**Affiliations:** 1 Department of Reproductive Medicine and Child Development, University of Pisa, Pisa, Italy; 2 Pathology Unit, University of Turin, Mauriziano “Umberto I” Hospital and Institute for Cancer Research and Treatment of Candiolo, Turin, Italy; 3 Department of Gynecological Oncology, University of Turin, Mauriziano “Umberto I” Hospital and Institute for Cancer Research and Treatment of Candiolo, Turin, Italy; University of Birmingham, United Kingdom

## Abstract

**Background:**

Estrogen is an established enhancer of breast cancer development, but less is known on its effect on local progression or metastasis. We studied the effect of estrogen receptor recruitment on actin cytoskeleton remodeling and breast cancer cell movement and invasion. Moreover, we characterized the signaling steps through which these actions are enacted.

**Methodology/Principal Findings:**

In estrogen receptor (ER) positive T47-D breast cancer cells ER activation with 17β-estradiol induces rapid and dynamic actin cytoskeleton remodeling with the formation of specialized cell membrane structures like ruffles and pseudopodia. These effects depend on the rapid recruitment of the actin-binding protein moesin. Moesin activation by estradiol depends on the interaction of ERα with the G protein Gα_13_, which results in the recruitment of the small GTPase RhoA and in the subsequent activation of its downstream effector Rho-associated kinase-2 (ROCK-2). ROCK-2 is responsible for moesin phosphorylation. The Gα_13_/RhoA/ROCK/moesin cascade is necessary for the cytoskeletal remodeling and for the enhancement of breast cancer cell horizontal migration and invasion of three-dimensional matrices induced by estrogen. In addition, human samples of normal breast tissue, fibroadenomas and invasive ductal carcinomas show that the expression of wild-type moesin as well as of its active form is deranged in cancers, with increased protein amounts and a loss of association with the cell membrane.

**Conclusions/Significance:**

These results provide an original mechanism through which estrogen can facilitate breast cancer local and distant progression, identifying the extra-nuclear Gα_13_/RhoA/ROCK/moesin signaling cascade as a target of ERα in breast cancer cells. This information helps to understand the effects of estrogen on breast cancer metastasis and may provide new targets for therapeutic interventions.

## Introduction

One out of eight women develop breast cancer at some stage throughout life [1]. Despite the recent improvements in survival rates, many patients relapse, and the majority of these patients die for disseminated metastatic disease, which supports the need for new therapeutic strategies.

In the mammary gland, the sex steroid estrogen promotes breast growth and development at puberty and during the menstrual cycle and pregnancy [2]. In addition to these physiological effects, estrogen plays a major role in the development and progression of breast cancer. Prolonged exposure to estrogen, i.e. early menarche, late menopause or postmenopausal hormone replacement therapy, is associated with a greater risk of developing breast cancer [3]. Estrogen promotes breast cancer proliferation through a number of established pathways [4]. On the contrary, the effects of estradiol on tumor cell motility and invasion are poorly understood.

Cell migration is required for cancer cell spread, invasion and metastasis and it is achieved through a dynamic remodeling of filamentous actin and of focal adhesion sites [5]. This process leads to rapid changes of cell membrane morphology, with the formation of specialized structures linked to cell movement such as pseudopodia and ruffles [6]. Estrogen administration to breast cancer cells is associated with estrogen receptor-α (ERα) membrane translocation and with the rapid formation of such specialized cell membrane structures [7]. Similar effects are found in human endothelial cells, where estrogen alters the cytoskeleton and increases cell migration through the activation of the actin binding protein, moesin [8].

Moesin belongs to the ezrin-radixin-moesin (ERM) family of actin-binding proteins [9]. By interacting with actin, activated ERMs induce actin de-polymerization and re-assembly toward the cell membrane edge, supporting the formation of cortical actin complexes [10]. These complexes help the formation of molecular bridges between the actin cytoskeleton, integrins and focal adhesion complexes within ruffles and pseudopodia and are critical for cell movement [6].

Ezrin, another member of the ezrin/radixin/moesin (ERM) family, is over-expressed in highly aggressive sarcomas [11], [12], as well as in breast cancer, being associated with higher metastasis rate [13]. In addition, ezrin expression is induced by estrogens in ovarian cancer cells [14] suggesting that ERM proteins might have important functions in cancer progression and/or metastasis.

In this paper we study the effects of estradiol on the migration and invasion of ER+ or ER− breast cancer cells and we relate these observations to actin remodeling and to the activation of moesin, characterizing the signaling steps involved in these actions. In addition, we study the expression and sub-cellular localization of wild type and activated moesin in normal breast tissue, benign breast disorders as well as in ER+ and ER− invasive breast carcinomas, highlighting the relationship with lymph node metastasis.

## Results

### Estrogen receptor activation induces a rapid cytoskeletal rearrangement and the development of specialized membrane structures

**Figure 1:**
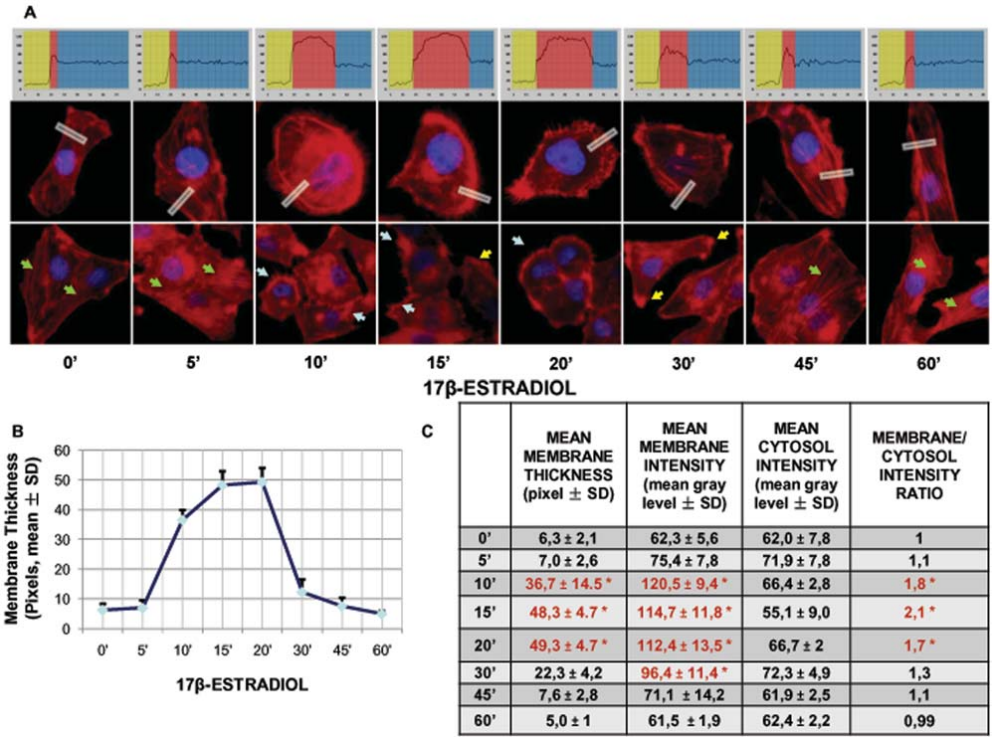
Recruitment of ER induces a dynamic remodeling of the actin cytoskeleton and of the cell membrane in T47-D breast cancer cells. A) T47-D cells were treated for different times with E2 (10 nM). Actin fibers were stained with phalloidin linked to Texas Red (red labeling) and nuclei were counterstained with DAPI (blue labeling). Immunofluorescent analysis reveals the dynamic modifications of actin fibers through the time-course and the formation of specialized cell membrane structures. Green arrows indicate longitudinal fibers; light blue arrows indicate membrane ruffles; yellow arrows indicate pseudopodia. Rectangles indicate the analyzed area which was demonstrated by the corresponding upper graph. In the graph, the longitudinal axis represents the gray level and the horizontal axis indicates pixels. Yellow, red and blue areas indicate the extracellular, plasma membrane and cytoplasmic fraction, respectively. B and C) Analytic results obtained by using Leica QWin image analysis and processing software showing the mean thickness of the cell membrane as well as the intensity of actin staining in the cytoplasm, membrane or extracellular compartment after treatment with E2 (10 nM) for different times. The results are derived from the sampling of five areas of the cell membrane of thirty different random cells. The areas of minimum and maximum cell membrane thickness were always included. The results are the mean±SD of the measurements. In C) * = p<0.05 vs. Basal condition (0 min).

To assess the potential effect of estrogen receptor (ER) recruitment on breast cancer cell movement, we studied the morphological changes of the actin cytoskeleton in ER+ T47-D cells exposed to estradiol. Unstimulated cells displayed mainly longitudinally-arranged actin fibres in the cytoplasm (Fig. 1A). Recruitment of ER with 17β-estradiol (E2, 10 nM) resulted in a rapid change in actin organization, with a remodeling of the fibres toward the cell membrane edge (Fig. 1A). Plus, exposure to E2 was associated with the formation of specialized membrane structures linked to cell attachment to the extracellular matrix and to cell movement, such as pseudopodia and membrane ruffles (Fig. 1A). This phenomenon was time-dependent and transient, being maximal after 15–20 minutes and then progressively reversing to the basal arrangement between 30 and 60 minutes (Fig. 1A). The remodeling of actin fibres and the morphological changes of the membrane were quantified by assessing the intensity of the actin fluorescence after conversion of the coloured pixels to greyscale using the Leica QWin image analysis and processing software. This analysis was performed selecting random boxes including the extra- and intra-cellular space across the membrane, and the linear intensity of the signal was spatially recorded. We sampled five areas per each cell, and we repeated this on 30 different cells per experimental condition. The upper graphs in Fig. 1A show the analysis of sample boxes (displayed in white in the lower images) used for the measurement. These measures allowed to identify the thickness of the membrane, that is shown throughout the time-course in Fig. 1B, as well as the mean±SD intensity of the actin staining in the membrane, in the cytosol, as well as the membrane/cytosol ratio (Fig. 1C).

### Estrogen receptor recruitment turns into a rapid activation of the actin-regulatory protein moesin

**Figure 2:**
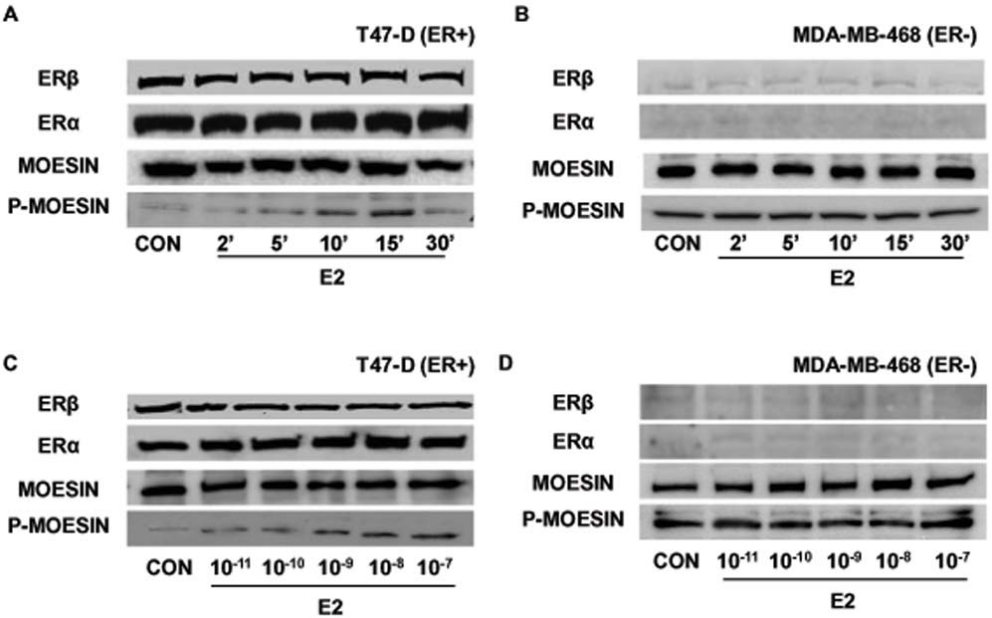
ER recruitment results in the activation of the actin-binding protein, moesin. Protein extracts from T47-D (A and C) and MDA-MB-468 (B and D) breast cancer cells treated for different times with 10 nM E2 (A and B) or for 15 min with increasing concentrations of E2 (C and D) were assayed with western analysis for their overall content of ERα and ERβ , wild type moesin (moesin) or Thr^558^-phosphorylated moesin (P-moesin).

Activation of ER with E2 turned into a rapid increase of Thr^558^ phosphorylation (which corresponds to activation) [15], [16] of the actin-binding protein moesin (Fig. 2A). The activation of moesin by E2 follows the same kinetics as actin rearrangement (Fig. 2A) and was achieved through extra-nuclear pathways, as no changes of cell immunoreactive moesin were found (Fig. 2A). In addition, ERα and ERβ were both expressed and their levels did not vary throughout the time-course (Fig. 2A). In estrogen-receptor negative MDA-MB-468 breast cancer cells moesin was constitutively phosphorylated and exposure to estradiol did not alter this activation any further, suggesting that this protein may be basally over-active in these cells (Fig. 2B). As expected, nearly undetectable ERα and ERβ expression was present in this cell line (Fig. 2B).

In T47-D breast cancer cells, the activation of moesin was related to the concentration of E2 and was triggered by physiological amounts of the steroid (Fig. 2C), whereas in MDA-MB-468 cells no change in moesin phosphorylation was detected throughout the range of estradiol concentrations (Fig. 2D).

### Moesin is required for estrogen receptor-induced actin remodeling

**Figure 3:**
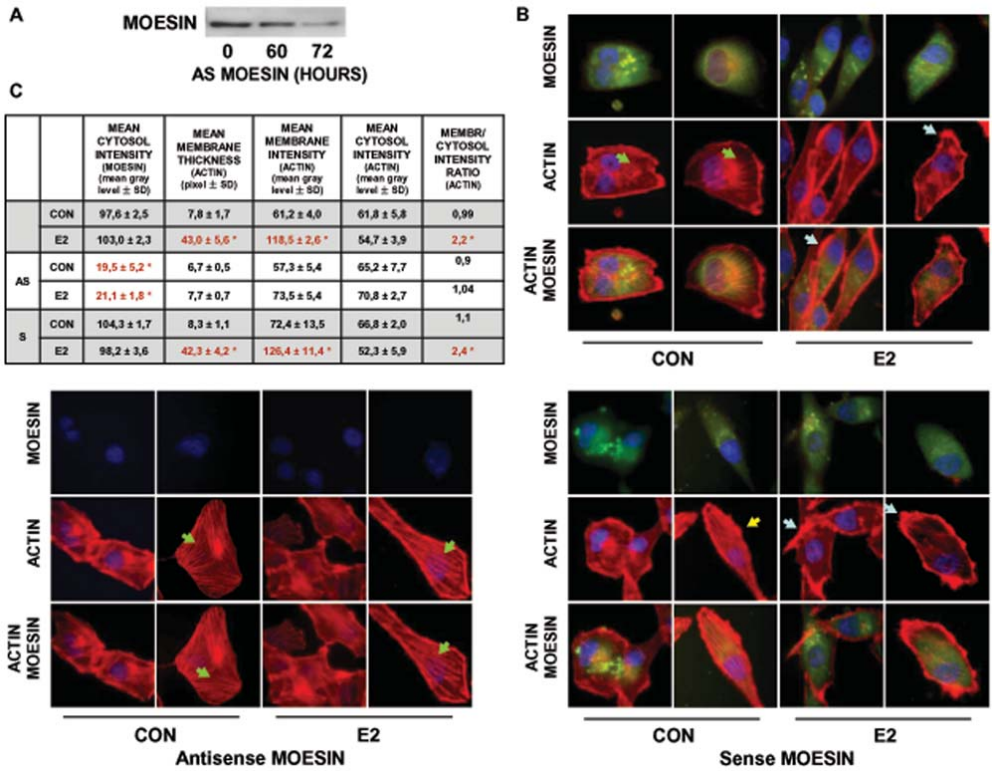
Moesin is required for estrogen-induced cytoskeletal remodeling. A) T47-D cells were transiently transfected with antisense (2 µM antisense MOESIN) moesin phosphorotioate oligonucleotides (PONs) for 0, 60 or 72 hours and moesin expression was assayed with western analysis. B and C) T47-D cells were transiently transfected with vehicle or antisense (2 µM antisense MOESIN) or sense (2 µM sense MOESIN) moesin phosphorotioate oligonucleotides (PONs) for 72 hours and then treated for 15 min with 10 nM E2. Moesin expression was checked by staining with a specific Ab linked to FITC (green labeling). Actin fibers were stained with phalloidin-Texas Red (red labeling) and nuclei were counterstained with DAPI (blue labeling). F) shows the analytic results obtained by using Leica QWin image analysis and processing software showing the mean thickness of the cell membrane as well as the intensity of actin and moesin staining in the cytoplasm, membrane or extracellular compartment. The results are derived from the sampling of five areas of the cell membrane of thirty different random cells. The areas of minimum and maximum cell membrane thickness were always included. The results are the mean±SD of the measurements. * = p<0.05 vs. vehicle-transfected control.

To assess whether moesin is required for the estrogen receptor-dependent cytoskeletal rearrangement in T47-D cells, we silenced moesin by transfection of specific antisense phosphorotioate oligonucleotides (PONs) and observed the actions of E2 on actin rearrangement. Moesin expression was significantly reduced when T47-D breast cancer cells were transfected with antisense moesin PONs for 72 h (Fig. 3A). In moesin-silenced cells, E2 failed to induce a rapid actin reorganization as well as to to increase membrane thickness or the actin membrane/cytosol ratio (Fig. 3B–C). Furthermore, moesin-silenced cells did not develop specialized membrane structures in the presence of E2 (Fig. 3B). As control, E2 was fully effective in cells transfected with sense (inactive) moesin PONs (Fig. 3B–C).

### Estrogen activates moesin via ERα

**Figure 4:**
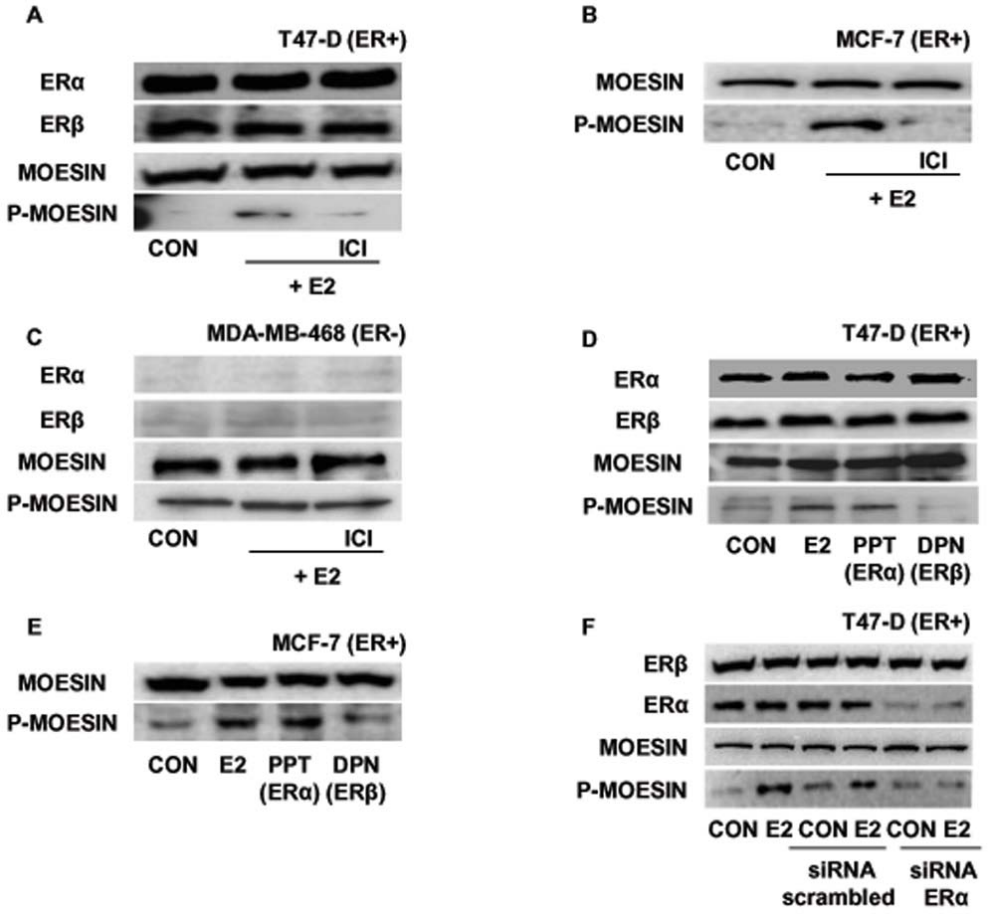
Estrogen signals to moesin via ERα. T47-D (A and D) , MCF-7 (B and E) or MDA-MB-468 (C) cells were treated for 15 minutes with either E2 (10 nM), the preferential ERα agonist PPT (10 nM) or the preferential ERβ agonist, DPN (10 nM), in the presence or absence of the ER antagonist, ICI 182,780 (1 µM). Western analyses of ERα, ERβ, wild-type moesin (moesin) or Thr^558^-phosphorylated moesin (P-moesin) were performed. F) T47-D cells were transfected with siRNAs vs. ERα (siRNA ERα), with scrambled siRNAs or with vehicle, and protein analysis for ERα, ERβ, wild-type moesin (moesin) or Thr^558^-phosphorylated moesin (P-moesin) was performed on cell lysates after treatment for 15 min with vehicle or 10 nM E2.

Moesin activation induced by E2 in T47-D cells was prevented by the addition of the pure ER antagonist ICI 182,780 (ICI, 1 µM) (Fig. 4A), indicating that ER is required. Since ICI 182,780 is reported to increase ER degradation, we checked for the levels of ERα and β in our experimental conditions. After a total exposure of 45 minutes to ICI (30 minutes of pre-treatment and 15 minutes of combined E2 and ICI 182,780 administration), we found no change in the levels of ERα or ERβ. Indeed, in T47-D cells, we find degradation of ERα in the presence of ICI 182,780 being visible only at time-points later than 60 minutes (data not shown). This is consistent with previous publications[17].

A similar ER-dependent activation of moesin was seen in MCF-7 cells (Fig. 4B). On the contrary, ICI 182,780 had no inhibitory effect on moesin phosphorylation in ER- MDA-MB-468 cells, supporting the concept that in these cells the recruitment of this protein does not require the presence of ERs (Fig. 4C).

To identify which ER isoform is required for the signaling of estrogen to moesin, we treated T47-D cells with estradiol or with the preferential ERα agonist 4,4′,4″-(4-propyl-[1H]-pyrazole-1,3,5-triyl)*tris*phenol (PPT, 1nM) [18] or with the ERβ agonist 2,3-*bis*(4-hydroxyphenil)-propionitrile (DPN, 1nM) [19]. Moesin activation was detected only in the presence of estradiol or PPT, suggesting that ERα supports the signaling to moesin, while ERβ is not required (Fig. 4D). In agreement, also MCF-7 cells treated with PPT or DPN showed moesin activation in the presence of the preferential ERα agonist (Fig. 4E).

Since the ER isoform selectivity of PPT and DPN is not absolute (at higher concentrations they bind to some extent both ERα and ERβ), we silenced ERα in T47-D cells with targeted small interfering RNAs (siRNAs). Transfection of ERα siRNAs resulted in a clear reduction of ERα expression, along with a dramatic decrease of the phosphorylation of moesin during exposure to estrogen (Fig. 4F). This happened in the absence of modifications of the expression of moesin or ERβ (Fig. 4F), supporting the hypothesis that ERα drives the signaling of estradiol to moesin.

### ERα signals to moesin through a Gα_13_- and RhoA-dependent signaling pathway

**Figure 5:**
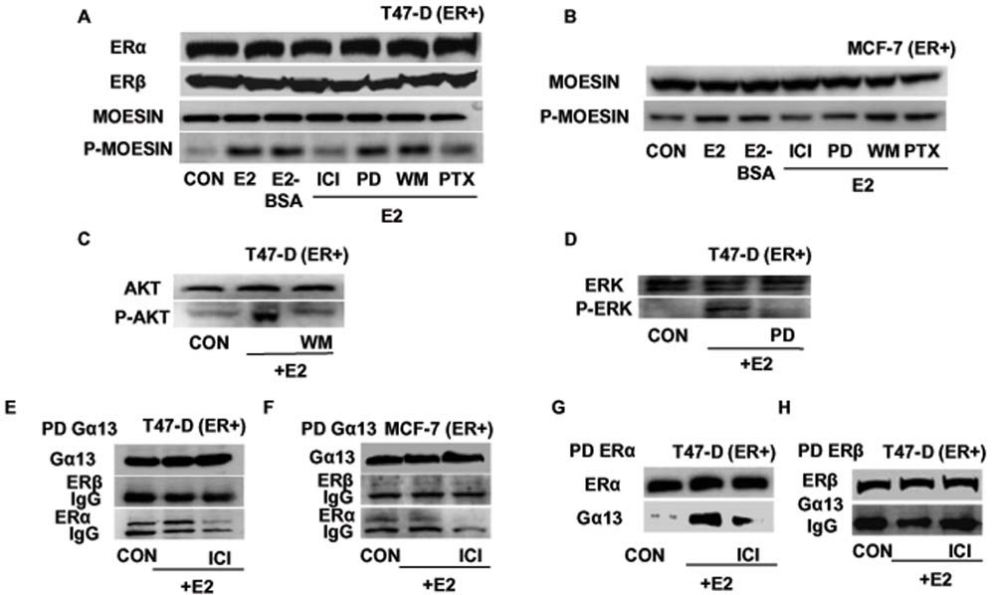
ERα signals to moesin via Gα_13_ and RhoA. T47-D (A) or MCF-7 (B) cells were exposed for 15 min to 10 nM E2 or to E2 conjugated to bovine serum albumin (E2-BSA; 10 nM), in the presence or absence of the ER antagonist ICI 182,720 (ICI; 1 µM), of the MAPK inhibitor PD98059 (PD; 5 µM), of the PI3 kinase inhibitor wortmannin (WM; 30 nM) or of the G protein inhibitor, PTX (100 ng/ml). ERα, ERβ, wild-type moesin (moesin) or Thr^558^-phosphorylated moesin (P-moesin) were assayed in cell extracts. C and D) T47-D cells were treated for 15 min with 10 nM E2, in the presence or absence of the MAPK inhibitor PD98059 (PD; 5 µM) or of the PI3 kinase inhibitor wortmannin (WM; 30 nM). ERK 1/2 or Tyr^204^-phosphorylated-ERK 1/2, Akt or Ser^473^-phosphorylated Akt were assayed in cell extracts. T47-D cells E) or MCF-7 cells F) were treated for 15 min with 10 nM E2 in the presence or absence of ICI 182,720 (ICI, 1 µM). Cell protein extracts were immunoprecipitated with an Ab. vs. Gα_13_ and the IPs were assayed for co-immunoprecipitation of ERβ or ERα. The membranes were re-blotted for Gα_13_ to show equal input. G and H) T47-D cell extracts were immunoprecipitated with an Ab. vs. ERα (G) or vs. ERβ (H) and the IPs were assayed for co-immunoprecipitation of Gα_13_. The membranes were re-blotted for ER α or β to show equal imput.

In the search for the signaling pathways through which ERα leads to moesin activation in breast cancer cells, we used different pharmacological inhibitors linked to ERα or moesin to treat estrogen-receptor positive breast cancer cells. PD98059 (PD, 5 µM), an inhibitor of mitogen-activated protein kinases (MAPK) and wortmannin (WM, 30 nM), an inhibitor of phosphatidylinositol 3-OH kinase (PI3K), did not alter the estradiol-induced moesin phosphorylation in T47-D (Fig. 5A) or MCF-7 cells (Fig. 5B). As control, PD98059 and wortmannin were fully able to block the estradiol-dependent activation of ERK 1/2 (Fig. 5C) or of the PI3K effector Akt (Fig. 5D) in T47-D breast cancer cells.

Instead, the G protein inhibitor, pertussis toxin (PTX, 100 ng/mL) significantly inhibited moesin phosphorylation induced by E2 (Fig. 5A and 5B). Moreover, the membrane-impermeable estradiol-bovine serum albumin conjugate (E2-BSA, 10 nM) triggered moesin activation similar to normal E2 (Fig. 5A and 5B). These findings suggest that a G protein-dependent, cell-membrane initiated mechanism mediates the signaling of ERα to moesin and to the actin cytoskeleton.

We previously showed that ERα is able to interact with the G protein, Gα_13_, that controls the small GTPase RhoA and its effector, Rho-associated kinase (ROCK) [8]. This signaling cascade is implicated in the control of the cytoskeleton in human vascular cells [8]. With co-immunoprecipitation assays we found that in T47-D as well as in MCF-7 cells estradiol triggers a direct association of ERα with Gα_13_ which is prevented by ICI 182,780 (Fig. 5E and 5F). In parallel, no co-immunoprecipitation of ERβ with Gα_13_ was found in either cell lines (Fig. 5E and 5F). Reverse co-immunoprecipitations of ERα (Fig. 5G) and ERβ (Fig. 5H) followed by western analysis of Gα_13_ confirmed that Gα_13_ and ERα (but not ERβ) co-interact in a ligand-dependent fashion in T47-D cells.

**Figure 6:**
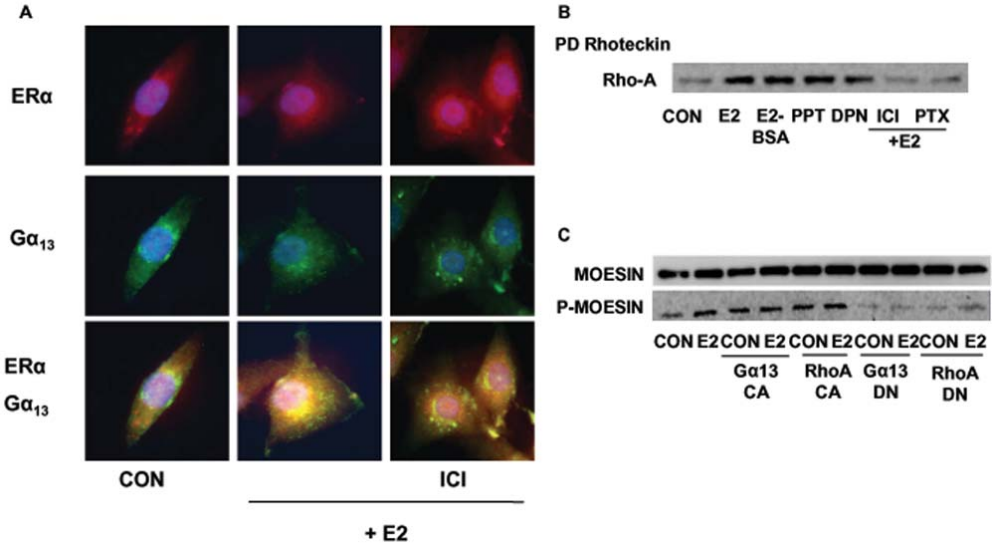
ERα signals to moesin via Gα_13_ and RhoA. A) T47-D cells were treated for 15 min with 10 nM E2 in the presence or absence of ICI 182,720 (ICI, 1 µM) and cells were stained with Abs vs. ERα (red staining) and Gα_13_ (green staining). Double staining, showing co-localization is presented in the lower boxes in yellow. B) RhoA activity was assayed in T47-D treated for 15 min with 10 nM E2, E2 conjugated to BSA (E2-BSA, 10 nM), the ERα-preferential ligand PPT (10 nM) or the ERβ-preferential agonist DPN (10 nM) in the presence or absence of ICI 182,720 (ICI, 1 µM) or PTX (100 ng/ml). Active, GTP-bound, RhoA was precipitated with Rhotekin, and a western analysis for RhoA was then performed. C) Whole-cell extracts were assayed for wild-type (moesin) or for Thr^558^-phosphorylated (P-moesin) moesin content after transfection with empty vector or with Gα_13_ or RhoA constitutively active (Gα_13_ CA and RhoA CA) or dominant-negative (Gα_13_ DN and RhoA DN) constructs, in the presence or absence of E2 (10 nM; 15 min).

With a double Immunofluorescent staining we were further able to show that in the presence of estradiol Gα_13_ and ERα partially co-localize in T47-D cells. In particular, spot-like double staining of Gα_13_ and ERα was found on the cell membrane in the presence of E2, which was antagonized by co-administration of ICI 182,780 (Fig. 6A).

By assaying RhoA GTP-binding activity, we found that RhoA is activated in T47-D cells by rapid exposure to estradiol or to the membrane-impermeable estradiol-BSA (Fig. 6B). RhoA activation was prevented by ER antagonism with ICI 182,780 and by interference with G proteins using PTX (Fig. 6B). The ERα-selective agonist PPT was associated with a strong activation of RhoA (Fig. 6B), while the ERβ-preferential ligand, DPN, was also found to increase RhoA GTP-binding, although to a lesser extent (Fig. 6B).

To further test the requirement of Gα_13_ and RhoA for ERα-induced moesin activation in T47-D breast cancer cells, we performed transient transfections with Gα_13_ (Gα_13_ Q226L) or RhoA (RhoA G14V) constitutively active mutated constructs or with Gα_13_ (Gα13 Q226L/D294N) or RhoA (RhoA T19N) dominant negative constructs (Fig. 6C). Breast cancer cells transfected with constitutively active constructs showed an over-active moesin phosphorylation that was independent of estradiol (Fig. 6C). Instead, dominant negative Gα_13_ or RhoA constructs significantly inhibited the estradiol-induced moesin activation (Fig. 6C). Overall, these results show that Gα_13_ and RhoA are implicated in the signaling of ERα to moesin in breast cancer cells.

### ERα and moesin phosphorylation: role of the Rho-associated kinase (ROCK-2)

**Figure 7:**
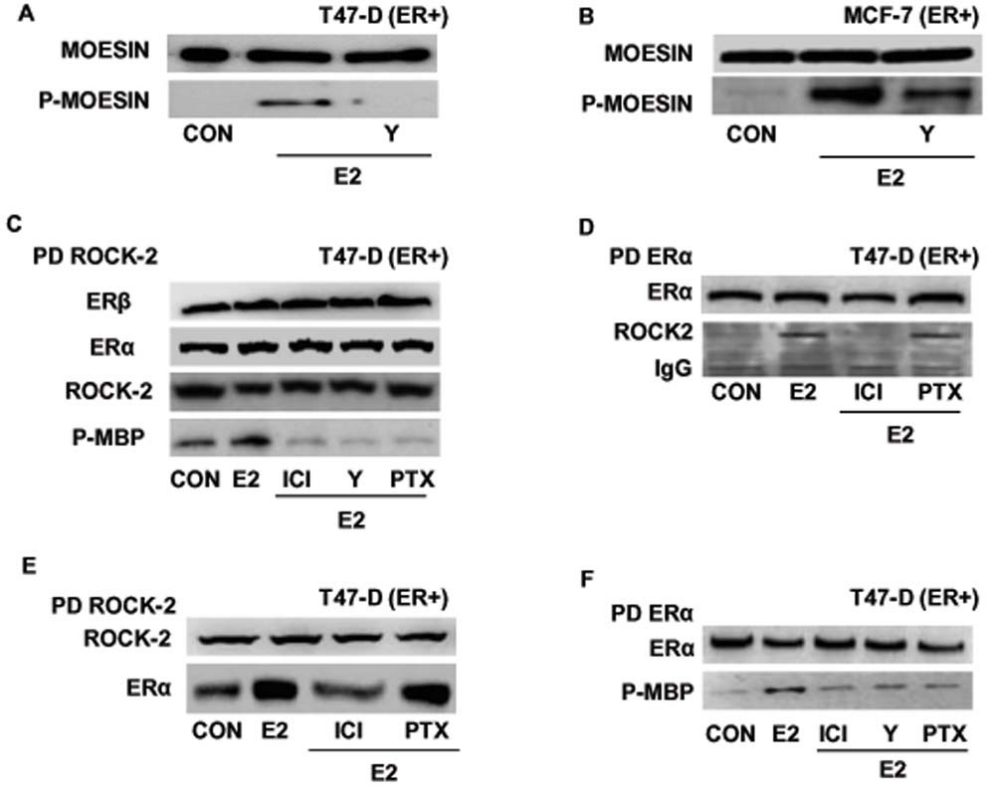
ERα recruits the Rho-associated kinase, ROCK-2. Breast cancer cells were treated for 15 min with 10 nM E2 in the presence or absence of the ROCK-2 inhibitor, Y-27632 (Y; 10 µM), ICI 182,720 (ICI; 1 µM), or PTX (100 ng/ml). Wild-type moesin (moesin) or Thr^558^-phosphorylated (P-moesin) moesin were assayed in T47-D (A) or MCF-7 (B) cell extracts. C) T47-D cancer cell protein extracts were immunoprecipitated with an Ab. vs. ROCK-2 and the IPs were used to perform kinase assays using de-phosphorylated myelin basic protein (MBP) as a bait. The lower box shows phosphorylation of MBP, the upper boxes show the re-blot of the membranes for ROCK-2, ERα or ERβ. D and E) T47-D cell protein extracts were immunoprecipitated with an Ab. vs. ERα (D) or ROCK-2 (E) and co-immunoprecipitation of ROCK-2 or ERα was assayed with western analysis. The membranes were re-blotted for ERα or ROCK-2 to show equal input. F) Breast cancer cell protein extracts were immunoprecipitated with an Ab. vs. ERα and the IPs were used to perform kinase assays using de-phosphorylated myelin basic protein (MBP) as a bait. The lower box shows phosphorylation of MBP, the upper box shows the re-blot of the membrane for the immunoprecipitated protein.

ROCK-2 phosphorylates moesin on Thr^558^ in different cell types, leading to filamentous actin fibers polymerization [20]. Pre-incubation of T47-D (Fig 7A) or MCF-7 (Fig 7B) breast cancer cells with the specific Rho-kinase inhibitor, Y-27632 (Y-27632, 10 µM) prevented the activation of moesin by estradiol, implying that ROCK-2 is required for estrogen signaling to moesin in ER+ breast cancer cells.

In agreement, ER activation resulted in increased ROCK-2 kinase activity in T47-D breast cancer cells (Fig. 7C). This was suppressed by ICI 182,780, Y-27632 as well as by PTX (Fig. 7C), indicating that ER recruitment turns into a G protein-dependent ROCK-2 activation.

In addition, we previously showed that ERα is able to interact with and directly activate ROCK-2 in human endothelial cells [8]. In T47-D breast cancer cells, an enhanced interaction between ERα and ROCK-2 was found with co-immunoprecipitation experiments in cells exposed to E2 (Fig. 7D–E). This protein-protein interaction was blocked by ICI 182,780 but not by PTX (Fig. 7D–E), suggesting that this may represent an additional mechanism for ERα-dependent ROCK-2 recruitment. To check whether the interaction with ERα turns ROCK-2 into an active status, we performed kinase assays using ERα immunoprecipitates (IPs) to target for Thr-phosphorylation the bait protein, myelin basic protein (MBP). ERα immunoprecipitates obtained from T47-D cells treated with estradiol induced MBP phosphorylation (Fig. 7F). ICI 182,780 and the ROCK-2 inhibitor Y-27632 counteracted this activation (Fig. 7F), confirming that ROCK-2 interacting with the ligand-engaged ERα is functionally activated. Consistently with the previous results, PTX did not reduce the activity of the ERα-associated-ROCK-2 (Fig. 7F), further suggesting the presence of two separate pathways of activation of ROCK-2 by ERα: one is G protein-dependent, while the second relies on a direct ERα/ROCK-2 interaction, which is not sensitive to PTX.

### Gα_13_, RhoA and ROCK-2 are necessary for the ER-dependent actin remodeling

**Figure 8:**
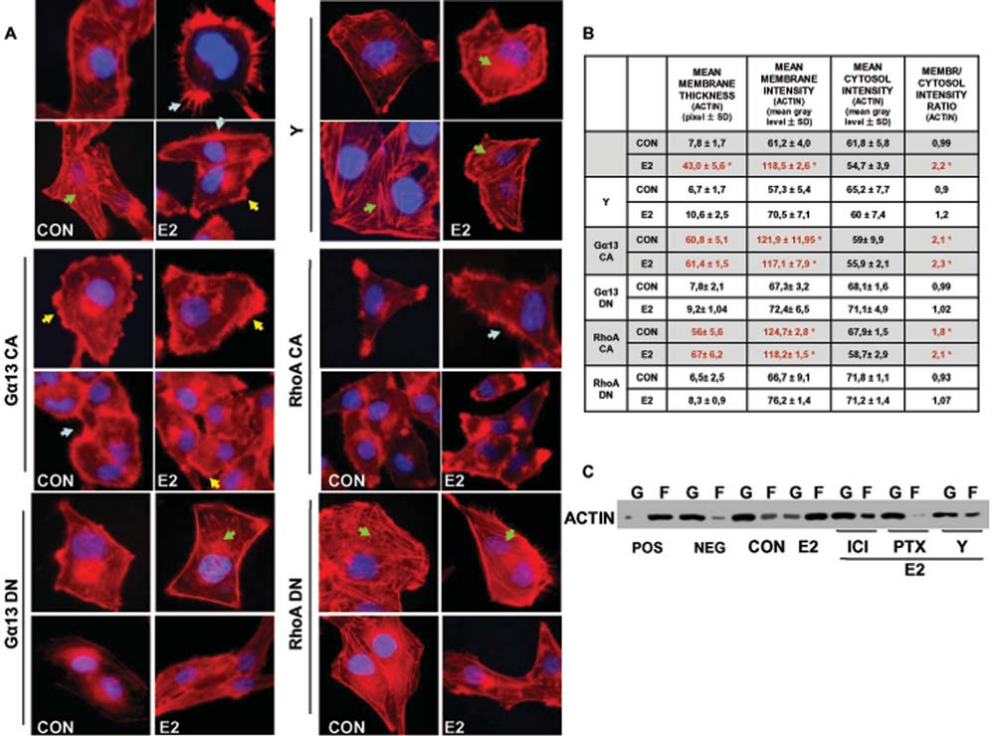
Actin remodeling by estrogen receptor requires Gα_13_, RhoA and ROCK-2. A) T47-D cells transiently transfected with either empty vector or with plasmids encoding for constitutively active or dominant-negative Gα_13_ (Gα_13_ CA or Gα_13_ DN) or RhoA (RhoA CA or RhoA DN) were treated for 15 min with 10 nM E2. Other cells also received a co-treatment with E2 (10 nM; 15 min) and the ROCK inhibitor Y-27632 (Y; 10 µM). Breast cancer cell actin fibers were stained with phalloidin linked to Texas Red, and nuclei were counterstained with DAPI. B) shows the analytic results obtained by using Leica QWin image analysis and processing software showing the mean thickness of the cell membrane as well as the intensity of actin staining in the cytoplasm, membrane or extracellular compartment. The results are derived from the sampling of five areas of the cell membrane of thirty different random cells. The areas of minimum and maximum cell membrane thickness were always included. The results are the mean±SD of the measurements. * = p<0.05 vs. vehicle-transfected control. C) shows the changes of the amount of filamentous actin (F-actin, F) content versus free globular-actin (G-actin, G) content in T47-D cells after treatment with E2 (10 nM) for 15 min, in the presence or absence of ER antagonist ICI 182,720 (ICI; 1 µM), G protein inhibitor PTX (100 ng/ml) and ROCK-2 inhibitor Y-27632 (Y; 10 µM). Positive (Pos) and negative (Neg) controls were set by adding F-actin enhancing solution (phalloidin, 1 µM) or F-actin depolymerization solution (10 µM cytochalasin-D) to the lysates, respectively.

To investigate whether Gα_13_, RhoA or ROCK-2 are required for the ER-induced cytoskeletal remodeling, we studied the architecture of actin fibers while transfecting T47-D cells with the Gα_13_ or RhoA mutated constructs or in the presence of the ROCK-2 inhibitor Y-27632. Both the Gα_13_ and RhoA constitutively active constructs triggered a visible actin cytoskeleton remodeling with generation of membrane protrusions, resulting in enhanced interaction of T47-D cells with the extracellular matrix and with nearby cells (Fig. 8A). Estradiol did not modify the cytoskeletal structure any further in these cells (Fig. 8A). In parallel, the dominant negative Gα_13_ and RhoA constructs abrogated the modifications of actin and cell morphology induced by estrogen (Fig. 8A). Moreover, the inhibition of ROCK with Y-27632 prevented the effect of estradiol on cytoskeleton remodeling (Fig. 8A). These changes were reflected in the measurements of the mean membrane intensity and actin membrane/cytosol ratio (Fig. 8B).

The changes in actin spatial organization were accompanied by parallel modifications of the ratio between globular and fibrillar actin. At baseline, actin fibers predominantly existed as monomers (G-actin) (Fig. 8C). After treatment with E2 for 15 min, a visible shift from G to F actin was seen (Fig. 8C), indicating that ER recruitment is linked to polymerization of G-actin into F-actin. The ER antagonist ICI 182,780, the G protein inhibitor, pertussis toxin (PTX) and the Rho-kinase inhibitor Y-12732 largely prevented the shift from G- to F-actin induced by estradiol (Fig. 8C).

### Recruitment of ER enhances breast cancer cell migration and invasion through a G protein/ROCK/moesin pathway

**Figure 9:**
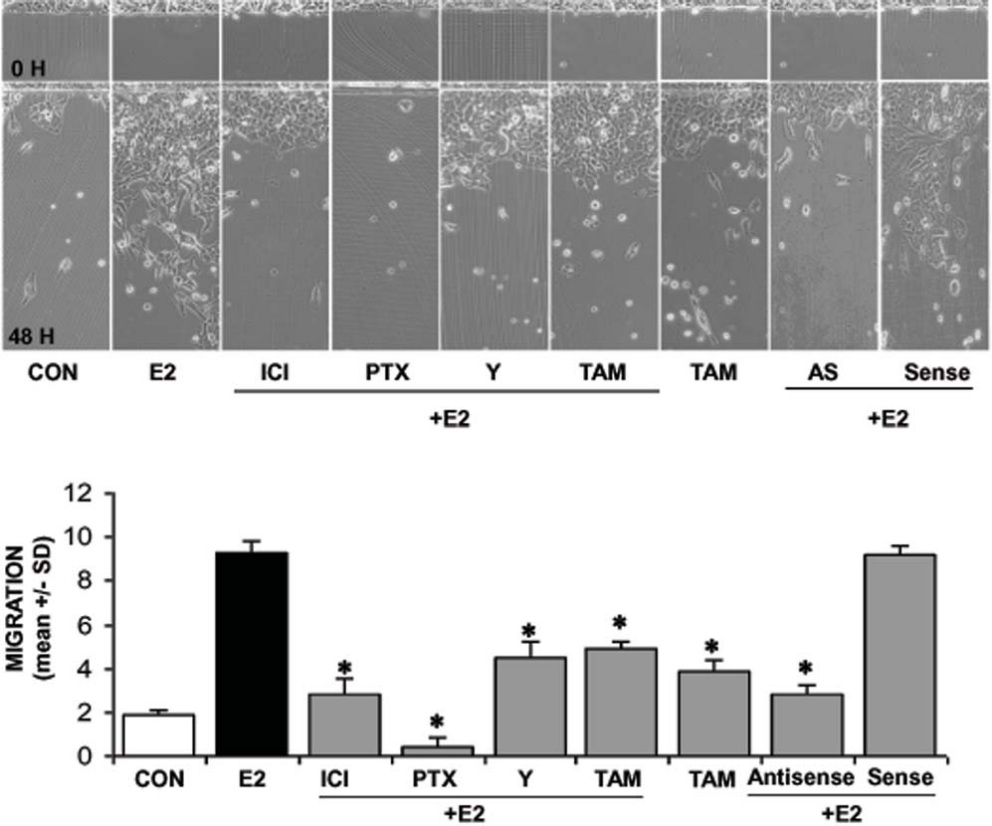
Role of the G protein/ROCK/moesin pathway for ER+ breast cancer cell migration. Steroid-deprived, growth synchronized ER+ T47-D cells were exposed to 10 nM E2, in the presence or absence of the ER antagonist ICI 182,720 (ICI; 1 µM), of the G protein inhibitor PTX (100 ng/ml), or of the ROCK-2 inhibitor, Y-27632 (Y; 10 µM) or of the selective estrogen receptor modulator (SERM) tamoxifen (TAM, 10 nM). In addition, some T47-D cells were treated with 10 nM E2 in the presence or absence of transfection with sense (Sense; 2 µM) or antisense (AS; 2 µM) moesin phosphorotioate oligonucleotides (PONs). Breast cancer cells were scraped from the culture dish and the mean migration distance of the cells from the starting line was assayed after 48 h and expressed as mean±SD. Representative images are shown and the mean±SD of migration are shown in the bar graphs. * = p<0.05 vs. E2.

To address the question of the relevance of the ERα/Gα_13_/RhoA/ROCK/moesin signaling cascade on breast cancer cell movement, we pretreated T47-D or MDA-MB-468 (ER-) breast cancer cells with cytosine arabinoside (1-(β-D-arabino­furanosyl)­cytosine hydrochloride-Ara-C, 100µM), an inhibitor of DNA strand separation that prevents cell division (so to dissect the actions of estrogen on movement from those on cell proliferation), and we performed horizontal migration assays. Estradiol (E2, 10 nM) strongly enhanced the migration of T47-D cells (Fig. 9). This was largely prevented by ICI 182,780, PTX and Y-27632, as well as by moesin silencing with antisense oligonucleotides (Fig. 9). In addition, the selective estrogen receptor modulator tamoxifen (TAM, 10 nM) that is used in the adjuvant treatment of ER+ breast cancers, also reduced the migration of T47-D cells induced by E2 while being ineffective on its own (Fig. 9).

**Figure 10:**
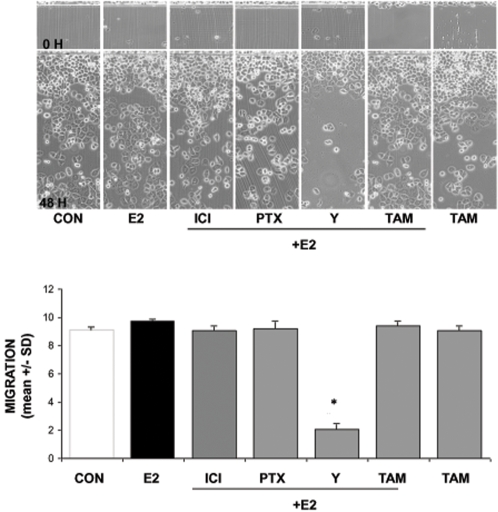
Role of the G protein/ROCK/moesin pathway for ER− breast cancer cell migration. ER− MDA-MB-468 cells were exposed to 10 nM E2, in the presence or absence of the ER antagonist ICI 182,720 (ICI; 1 µM), of the G protein inhibitor PTX (100 ng/ml), or of the ROCK-2 inhibitor, Y-27632 (Y; 10 µM) or of the selective estrogen receptor modulator (SERM) tamoxifen (TAM, 10 nM). Breast cancer cells were scraped from the culture dish and the mean migration distance of the cells from the starting line was assayed after 48 h and expressed as mean±SD. Representative images are shown and the mean±SD of migration are shown in the bar graphs. * = p<0.05 vs. control.

In general, ER- MDA-MB-468 cells showed a stronger tendency to migrate in comparison to T47-D cells and this was not affected by E2, ICI, PTX or tamoxifen (Fig. 10). However, the ROCK inhibitor, Y-27632, significantly decreased the migration of these cells (Fig. 10), suggesting that ROCK-2 may be overactive in MDA-MB-468 cells, and that this may enhance their ability to migrate.

**Figure 11:**
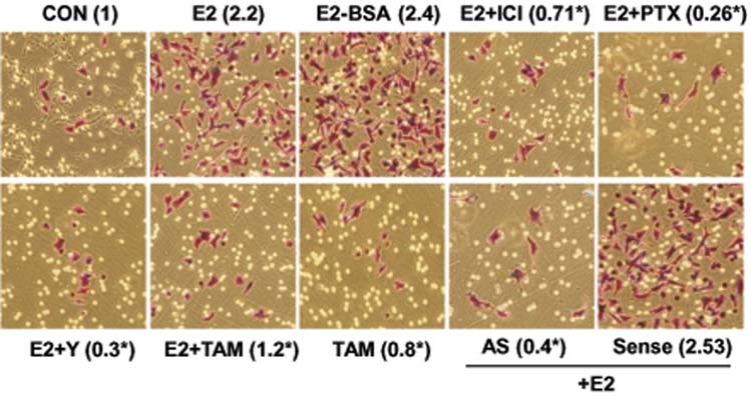
Role of the G protein/ROCK/moesin pathway for ER+ breast cancer cell invasion. Steroid-deprived, growth synchronized ER+ T47-D cells were exposed to 10 nM E2, or 10 nM E2-BSA conjugate in the presence or absence of the ER antagonist ICI 182,720 (ICI; 1 µM), of the G protein inhibitor PTX (100 ng/ml), or of the ROCK-2 inhibitor, Y-27632 (Y; 10 µM) or of the selective estrogen receptor modulator (SERM) tamoxifen (TAM, 10 nM). In addition, some T47-D cells were treated with 10 nM E2 in the presence or absence of transfection with sense (Sense; 2 µM) or antisense (AS; 2 µM) moesin phosphorotioate oligonucleotides (PONs). Breast cancer cell invasion through matrigel was assayed by using invasion chambers. Invading cells were counted in the central field of triplicate membranes. Invasion indexes and representative images in chambers with matrigel are shown. * = p<0.05 vs. E2.

To test the impact of the ER-dependent signaling to moesin on breast cancer cell invasion we performed three-dimensional invasion assays using matrigel. Ara-C-pretreated cells showed an enhanced invasion of the matrix in the presence of E2 or E2-BSA (Fig. 11). The effect of E2 was blocked by the ER-antagonist ICI 182,780, by PTX, by the ROCK-inhibitor Y-27632 as well as by tamoxifen (Fig. 11). In addition, transfection of moesin antisense PONs also resulted in impaired invasion in the presence of estradiol (Fig. 11).

### Moesin and P-moesin sub-cellular localization in normal breast tissue, benign breast disease and invasive breast cancer

To further characterize the biological role of moesin, we studied the sub-cellular distribution of wild-type moesin and activated moesin in normal and tumoral human breast tissues by immunohistochemistry.

**Figure 12:**
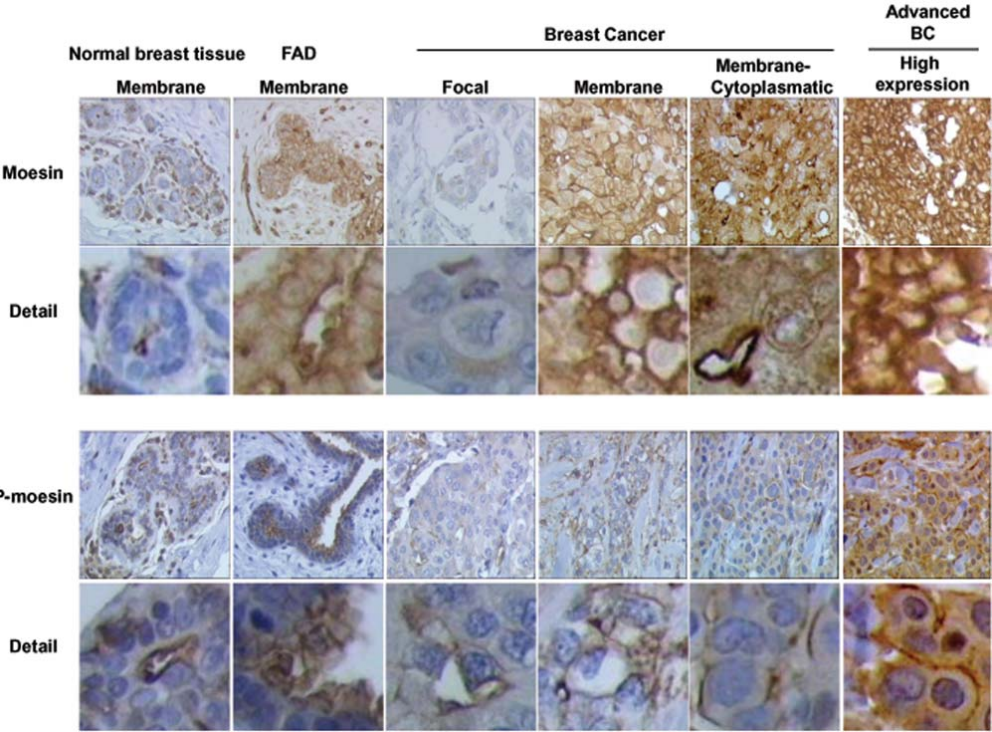
Moesin and P-moesin expression and sub-cellular localization in human normal breast tissue, benign fibroadenomas and breast cancers. Histological sections (4 µm) from normal mammary glands, fibroadenomas or ER positive (ER+) breast cancers were used to identify the expression and localization of moesin and P-moesin with immunochemistry. Wild-type moesin as well as Thr^558^-phosphorylated are shown as brown labeling. The figure displays sample images from the tumors analyzed in Table 1. Particularly, The normal breast tissue comes from Patient 2, the fibroadenomas is from patient 3. The breast cancer showing focal staining is that of patient 20, the cancer with membrane staining comes from patient 13. The cancer showing mixed membrane/cytoplasmic staining is from patient 17. The “high expression” cancer is from patient 12.

In normal human mammary ducts, staining for wild-type and phosphorylated moesin was found on the apical surface of ductal epithelial cells (Fig. 12), consistent with the usual localization in polarized secretory cells [9]. In addition, both moesin and P-moesin staining was present in occasional basal myo-epithelial elements and in endothelial cells (Fig. 12). Similar to normal breast tissue, in breast fibroadenomas (FAD) moesin and P-moesin were prominently located at the cell membrane of epithelial elements (Fig. 12), along with occasional stromal and myo-epithelial cells.

In addition, in order to characterize moesin and P-moesin expression and distribution in invasive breast cancers, we stained samples from human ductal carcinomas (tumor stage: pT1c–node size between 1 and 2 cm-see Table 1) and we compared the pattern of staining of ER positive or negative cancers, with or without lymph node metastasis.

**Table 1:** 

NORMAL BREAST											
				MOESIN	MEM	MEM	CYTO	P-MOESIN	MEM	MEM	CYTO
						CYTO				CYTO	
1				INTENSE	**x**			INTENSE	**x**		
2				INTENSE	**x**			INTENSE	**x**		
3				INTENSE	**x**			INTENSE	**x**		
4				INTENSE	**x**			INTENSE	**x**		
5				INTENSE	**x**			INTENSE	**x**		
**FIBROADENOMAS**											
				**MOESIN**	MEM	MEM	CYTO	**P-MOESIN**	MEM	MEM/CITO	CYTO
						CYTO					
6				INTENSE	**x**			INTENSE	**x**		
7				INTENSE	**x**			INTENSE	**x**		
8				INTENSE	**x**			INTENSE	**x**		
9				INTENSE	**x**			INTENSE	**x**		
10				INTENSE	**x**			INTENSE	**x**		
**INV. DUCTAL CARCINOMAS**											
	SIZE	pNx	ER%	**MOESIN**	MEM	MEM	CYTO	**P-MOESIN**	MEM	MEM	CYTO
						CYTO				CYTO	
**ER POS/LYMPH NODE POS**											
11	18 mm	pN2a (7/16)	84%	FOCAL	**x**			NEGATIVE			
12	19 mm	pN1m (1/8)	98%	INTENSE			**x**	INTENSE	**x**		
13	15 mm	pN1a (1/3)	98%	INTENSE	**x**			INTENSE	**x**		
14	16 mm	pN1a (2/24)	100%	INTENSE		**x**		FOCAL			**x**
15	16 mm	pN3a (8/15)	95%	INTENSE		**x**		FOCAL			**x**
**ER POS/LYMPH NODE NEG**											
16	14 mm		90%	INTENSE		**x**		FOCAL			**x**
17	15 mm		90%	INTENSE		**x**		INTENSE		**x**	
18	10 mm		100%	INTENSE	**x**			INTENSE		**x**	
19	15 mm		99%	FOCAL	**x**			INTENSE	**x**		
20	18 mm		98%	FOCAL	**x**			FOCAL	**x**		
**ER NEG/LYMPH NODE POS**											
21	18 mm	pN2a (7/16)	0%	FOCAL			**x**	NEGATIVE			
22	17 mm	pN1m (1/17)	0%	INTENSE			**x**	INTENSE		**x**	
23	11 mm	pN1m (1/1)	0%	INTENSE		**x**		INTENSE	**x**		
24	17 mm	pN1a (2/19)	0%	INTENSE			**x**	FOCAL			**x**
25	13 mm	pN3b (20/24)	0%	INTENSE		**x**		FOCAL		**x**	
**ER NEG/LYMPH NODE NEG**											
26	8 mm		0%	INTENSE		**x**		NEGATIVE			
27	16 mm		0%	INTENSE		**x**		FOCAL			**x**
28	13 mm		0%	INTENSE		**x**		FOCAL	**x**		
29	15 mm		0%	INTENSE			**x**	NEGATIVE			
30	12 mm		0%	FOCAL		**x**		INTENSE	**x**		
**ALL ER NEG TUMORS VS. ALL ER POS TUMORS P<0.05 FOR ABSENCE OF MEMBRANE LOCALIZATION**											

Immunohistochemical analysis of moesin and P-moesin in normal breast tissue, benign breast disease and breast cancers. Negative indicates visible staining in less than 1% of the cells. Focal indicates staining in less than 30% cells. Intense indicates diffused staining throughout the tissue. Membrane (MEM) indicates staining exclusively localized to the cell membrane in more than 90% of the cells; membrane/cytoplasm (MEM/CYTO) indicates staining both on the cell membrane as well as in the cytoplasm in more than 90% of the cells; cytoplasm (CYTO) indicates staining of the cytoplasm without any evident membrane staining in more than 90% of the cells. Staining patterns were independently adjudicated by two pathologists. SIZE indicates the diameter of the mammary node. pNx indicates the number of positive lymph nodes. ER% indicates the percentage of tumor cells expressing ERs.

Different from normal breast tissues or fibroadenomas, invasive ductal carcinomas showed a deranged moesin and P-moesin cellular localization, with four main expression/distribution patterns. Some cancers showed a weak, focal expression of moesin (focal, less than 30% of the cells stain for moesin, Fig. 12). Other cancers instead showed an intense staining for moesin, but some had a strong expression of moesin at the cell membrane (Fig. 12), while others showed a mixed membrane/cytoplasmic staining (Fig. 12). Other tumors had instead a clear cytoplasmic moesin staining, in the absence of membrane localization (Fig. 12).

There was no significant statistical difference in the association of the type of staining with lymph node metastasis status in ER+ or ER− cancers. However, ER− tumors showed a statistically significant difference vs. ER+ cancers in wild-type moesin localization, having consistently no membrane-only staining while displaying a constant cytoplasmic moesin localization (Table 1). In addition, although this was not significant, ER− cancers with lymph node metastasis tended to display more frequently a cytoplasmic-only moesin staining, without any membrane localization (Table 1).

When P-moesin staining was studied, similar patterns of sub-cellular distribution were found in invasive cancers (focal vs. intense staining; membrane vs. membrane/cytoplasmic vs. cytoplasmic, Fig. 12), but there was no significant association with either ER or lymph node status (Table 1).

## Discussion

Estrogens act as promoters of cell proliferation and movement in different tissue types, including the breast [2]. This action is particularly relevant in the presence of estrogen receptor positive (ER+) breast cancers, that are driven to grow, invade and metastasize by endogenous or exogenous estrogens [4], [21]. For these reasons, understanding the basis through which estrogens drive cancer cells to interact with the extracellular environment to enact movement and invasion heralds profound biological and medical implications.

We here show that estrogen enhances horizontal migration and invasion of three-dimensional matrices of ER+ breast cancer cells by recruiting the actin-binding protein, moesin. Moesin resides at the nexus of multiple pathways regulating cell attachment with the extracellular matrix and with nearby cells, cell motility and metastatic potential as well as cell survival. These functions are directed by moesin though the modulation of actin cytoskeleton/plasma membrane interactions.

Moesin activation by estrogen in breast cancer cells is linked to the rapid and dynamic remodeling of the actin cytoskeleton and to the formation of specialized cell membrane structures, such as pseudopodia and ruffles, that are involved in the interaction with the extracellular matrix and required for cell movement [6], [22]. These actions are consistent with our previous observation of improved wound healing due to enhanced endothelial cell movement in the presence of estrogens, linked to the activation of moesin [8], as well as with the recently reported activation of cytoskeletal remodeling and cell migration by estrogens in endometrial cancer [5].

Our findings also strengthen the concept that the nongenomic signaling of estrogen receptor to ERM proteins and actin may be of general relevance for the determination of cell movement. Indeed, the actions described in this report occur within minutes without the need of activating gene expression [23] and are therefore dynamically shut off within 30 to 60 minutes. This makes sense in light of the established requirement of multiple, periodic, waves of actin remodeling and dynamic formation of anchorage sites to the extracellular matrix in order to accomplish cell movement [6], [22].

In addition, loss of stress fibers is associated with cancer cell transformation and metastasis [24]. Thus, the cytoskeletal rearrangement induced by estrogen through moesin may in part explain the carcinogenic actions of this steroid in estrogen-sensitive breast cancers, along with the enhanced metastatic behavior of such neoplasms in the presence of sex steroid hormones [4]. In support of this, similar actions of estrogen linked to the regulation of the ERM protein ezrin have been described in estrogen-sensitive cancers [14], [25].

ER- breast cancers, such as MDA-MB-468 cells, are not sensitive to estrogen administration in terms of moesin activation and cell migration. However, these cells display a basal hyper-activation of moesin. In addition, a direct inhibitor of moesin phosphorylation (ROCK inhibitor) is highly effective in reducing MDA-MB-468 cell horizontal migration. These findings are intriguing, as they suggest that sustained moesin activation by ROCK might be an important player in the metastatic behavior of ER- breast cancer cells. This fits with a previous report of a prominent expression of moesin in ER- human breast tumors, related to the tendency to metastasize [26].

The mechanistic basis that supports estrogen signaling to moesin and the actin cytoskeleton is found in the recruitment of the Gα_13_/RhoA/ROCK pathway by ERα. Similarly to what previously found in human endothelial cells [8], estrogen-bound ERα dynamically interacts with the G protein Gα_13_ and triggers its activation. This leads to the recruitment of the small GTPase RhoA and of its downstream effector, ROCK-2, which is responsible for moesin phosphorylation. These signaling intermediates, including moesin, are all required for actin remodeling as well as breast cancer cell migration and invasion. Interestingly, ERβ does not support the interaction with Gα_13_ nor it activates moesin. Overall, this further establishes the nongenomic ERα/Gα_13_/RhoA/ROCK/moesin signaling cascade as an important controller of cell movement by estrogen in different cell types, including cancer cells.

We also find that human breast cancers display a deranged over-expression and over-activation of moesin respect to normal breast tissues and/or benign fibroadenomas. Although our observation is only descriptive and limited to a small sample of patients, it is remarkable to find some variations in the sub-cellular localization of moesin in different cancer types. For instance, we find a statistically significant loss of membrane localization of moesin in ER− vs. ER+ cancers, which calls for future work to further characterize if the overall expression of this protein or its sub-cellular positioning might be associated with different cancer biology or behavior. This would be consistent with what is already known for the parent ERM protein, ezrin. Ezrin has been found to be over-expressed in invasive epithelial neoplasia, such as in estrogen-sensitive endometrial [25] and breast carcinomas [13], [27], and in highly aggressive sarcomas as well [11], [12], being related in many of these studies to the presence of tumor metastasis.

In conclusion, the present results suggest that within the broader range of actions of estrogen receptors, the rapid extra-nuclear signaling to the actin cytoskeleton through the Gα_13_/RhoA/ROCK/moesin cascade is relevant for the determination of estrogen-dependent breast cancer cell movement and invasion that are related to cancer metastasis. The characterization of these actions increase our understanding of the effects of estrogens on breast cancer progression and might be useful to develop new tools to interfere with the ability to diffuse locally or at distant sites of ER+ or ER− breast carcinomas.

## Materials and Methods

### Cell cultures and treatments

The human breast carcinoma cell line T47-D, MCF-7 and MDA-MB-468 were obtained from the American Type Culture Collection. T47-D cells were grown in RPMI 1640 supplemented with L-glutamine (2mM), 10% fetal bovine serum. MCF-7 were cultured in minimal essential medium (MEM) with L-glutamine (2mM) and 10% fetal bovine serum. MDA-MB-468 cells were cultured in McCoy's 5A medium with L-glutamine (2mM) and 10% fetal bovine serum. Before treatments, all breast cancer cells lines were kept 24 hours in medium containing steroid-deprived FBS. Before experiments investigating non-transcriptional effects, cancer cells were kept in medium containing no FBS for 8 hours. Whenever an inhibitor was used, the compound was added 30 minutes before starting the treatments. 17β-estradiol, 17β-estradiol-BSA, tamoxifen, PTX, Y-27632, PD98059 and wortmannin were from Sigma-Aldrich (Saint-Louis, MO), ICI 182,780, PPT and DPN were obtained by Tocris Cookson (Avonmouth, UK).

### Immunoblottings

Cell lysates were separated by SDS-PAGE. Antibodies used were: moesin (clone 38, Transduction Laboratories, Lexington, KY), Thr^558^-P-moesin (sc-12895, Santa Cruz Biotechnology, Santa Cruz, CA), ERα (TE111, NeoMarkers, Union City, CA), ERβ (N-19, Santa Cruz), ROCK-2 (C-20, Santa Cruz), Gα_13_ (A-20, Santa Cruz), RhoA (26C4, Santa Cruz), wild-type ERK 1/2 (polyclonal, catalog no. 442704; Calbiochem Merck Biosciences, GmbH, Darmstadt, Germany), Tyr^204^-P-ERK 1/2 (polyclonal, catalog no. sc-7976; Santa Cruz Biotechnology, Santa Cruz, CA), wild-type and Ser^473^-phosphorylated Akt (Upstate Biotechnology, Inc., Lake Placid, NY). Primary and secondary Abs were incubated with standard technique. Immunodetection was accomplished with enhanced chemiluminescence.

### Cell immunofluorescence

T47-D cells were grown on coverslips and exposed to treatments. Cells were fixed and permeabilized with methanol at −20°C for 10 min. Blocking was performed with 3% normal serum for 20 min. Cells were incubated with antibodies against human moesin (clone 38, Transduction Laboratories) or with Texas Red-phalloidin (Sigma). The nuclei were counterstained with or 4′-6-diamidino-2-phenylindole (DAPI) (Sigma) and mounted with Vectashield mounting medium (Vector Laboratories, Burlingame, CA). Immunofluorescence was visualized using an Olympus BX41 microscope and recorded with a high-resolution DP70 Olympus digital camera. After conversion to greyscale images, the cell membrane thickness and the gray levels of the extracellular area, cell membrane as well as cytoplasm were quantified using the Leica QWin image analysis and image processing software (Leica Microsystems, Wetzlar, Germany).

### Co-immunoprecipitation assays

T47-D cells were harvested in 100 mM Tris-HCl, pH 6.8, 4% SDS, 20% glycerol, 1 mM Na_3_VO_4_, 1 mM NaF, and 1 mM PMSF. Equal amounts of cell lysates were incubated with 1 µg of precipitating Ab (ERα, ROCK-2 or Gα_13_) for 1 hour at 4°C under gentle agitation. 25 µL of a 1∶1 protein A-agarose slurry were added, and the samples were rolled at 4°C for another hour. The samples were then pelleted, washed, and resuspended in 50 µL of 2× Laemmli buffer for immunoblotting.

### Kinase assays

T47-D cells were harvested in 20 mM Tris-HCl, 10 mM EDTA, 100 mM NaCl, 0.5% IGEPAL, 0.1 mg/mL PMSF. Equal amounts of cell lysates were immunoprecipitated with an Ab vs. ERα or ROCK-2. The IPs were washed three times with 20 mM Tris-HCl,10 mM EDTA, 150 mM NaCl 0.1% IGEPAL, 0.1 mg/mL PMSF. Two additional washes were performed in kinase assay buffer (20 mM MOPS, 25 mM β-glycerophosphate, 5 mM EGTA, 1 mM DTT) and the samples were therefore resuspended in this buffer. 5 µg of de-phosphorylated myelin basic protein (Upstate, Lake Placid, NY) together with 500 µM ATP and 75 mM MgCl_2_ were added to each sample and the reaction was started putting the samples at 30°C for 20 min. The reaction was stopped on ice and by resuspending the samples in Laemmli Buffer. The samples were separated with SDS-PAGE and western analysis was performed using an Ab recognizing Thr^98^-P-myelin Basic Protein.

### Rho Activity Assay

T47-D cells were treated and harvested in Mg^2+^ Lysis/Wash Buffer (125 mM HEPES, pH 7.5, 750 mM NaCl, 5% Igepal CA-630, 50 mM MgCl_2_, 5 mM EDTA and 10% glycerol). 1 µg of proteins were used to pull-down active, GTP-bound, RhoA using Rhotekin-GSH-agarose. The IPs were washed with Mg^2+^ Lysis/Wash Buffer and therefore resuspended in 2× Laemmli buffer. The IPs were separated on a denaturing acrylamide gel and western analysis with an anti-RhoA Ab was performed.

### G-actin/F-actin assay

The G-actin/F-actin assay kit was purchased from Cytoskeleton Inc (# BK037, Denver, USA). This kit is used to determine accurately the amount of filamentous actin (F-actin) content versus free globular-actin (G-actin) content in a cell population. In brief, confluent T47-D cells were harvested with lysis at 37°C and F-actin stabilization buffer (50 mM PIPES, 50 mM KCl, 5 mM MgCl_2_, 5 mM EGTA, 5% glycerol, 0.1% Nonidet P40, 0.1% Triton X-100, 0.1% Tween 20, 0.1% 2-mercapto-ethanol, 0.001% antifoam C, 1 mM ATP) after treatments. Total protein concentration was determined by standard method. Positive and negative controls were set by adding F-actin enhancing solution (phalloidin, 1 µM) or F-actin depolymerization solution (10 µM cytochalasin-D) to the lysates, respectively. The lysates were incubated at 37°C for 10 min, followed by a centrifuge at 2000 rpm for 5 min to pellet and discard unbroken cells. Supernatant were centrifuged at 10,000 × *g* for 1 h at 37°C. After that, supernatant and pellet were both collected. Pellets were resuspended to the same volume as the supernatant using ice cold distilled water plus F-actin depolymerization solution (10 µM cytochalasin-D) and put on ice for 1 h to dissociate F-actin. According to the protein concentration previously measured, equivalent volumes of supernatant and dissolved pellet were loaded to run Western blot and G-actin/F-actin ratio was quantified using a quantitative digital imaging system.

### Transient transfections

T47-D cells were transfected with each plasmid (15 µg) using the Lipofectin reagent (Invitrogen, Carlsbad, CA) according to the manufacturer's instructions. The transfected plasmids were: Gα_13_ Q226L, Gα_13_ Q226L/D294N, RhoA T19 and RhoA G14V, Ras G12V and Ras S17N. All the inserts were cloned in pcDNA3.1+. The constructs were obtained from the Guthrie cDNA Resource Center (www.cdna.org). As control, parallel cells were transfected with empty pcDNA3.1+ plasmid. Cells (60–70% confluent) were treated 24 hours after transfection and cellular extracts were prepared according to the experiments to be performed.

### Gene silencing with RNA interference

Two synthetic small interfering RNA targeting estrogen receptor α (siRNA SMARTpool ESR1, Dharmacon, USA) were used at the final concentration of 100 nM to silence ERα according to the manufacturer's instructions. T47-D breast cancer cells were treated 60 hours after siRNA transfection. The efficacy of gene silencing was checked with western analysis and found to be optimal at 60 hours.

### Moesin silencing with antisense oligonucleotides

Validated antisense phosphorotioate oligonucleotides (S-modified) (PONs) complementary to the 1-15 position of the human moesin gene coding region [28] were obtained. The sequence was 5′-TACGGGTTTTGCTAG-3′ for moesin antisense PON. The complementary sense PON was used as control (5′-CTAGCAAAACCCGTA-3′). PONs transfections were performed on sub-confluent T47-D breast cancer cells. PONs were resuspended in serum-free medium with 2% Lipofectin (Invitrogen) and added to the culture medium every 12 hours at the final concentration of 4 µM. Every 24 hours T47-D cells were washed and fresh medium supplemented with 4 µM of PONs was added. Moesin silencing was assessed through protein analysis up to 72 hours following transfection.

### Cell migration assay

Cell migration was assayed with razor scrape assays. Briefly, a razor blade was pressed through the confluent T47-D breast cancer cell monolayer into the plastic plate to mark the starting line. T47-D cells were swept away on one side of that line. Cells were washed, and 2.0 mL of DMEM containing steroid-deprived FBS and gelatin (1 mg/mL) were added. Cytosine β-D-arabinofuranoside hydrochloride (Sigma) (10 µM), a selective inhibitor of DNA synthesis which does not inhibit RNA synthesis was used 1 h before the test substance was added to prevent cell proliferation. Absence of cell proliferation and viability of the cells were checked in preliminary experiments with MTT (3-(4,5-dimethylthiazol-2-yl)-2,5-diphenyltetrazolium bromide) tests. Migration was monitored for 48 hours. Every 12 h fresh medium and treatment were replaced. Cells were digitally imaged and migration distance was measured by using phase-contrast microscopy.

### Cell invasion assay

Cell invasion were assayed using the BD BioCoat™ Growth Factor Reduced (GFR) Matrigel™ Invasion Chamber (BD Bioscience, USA). In brief, after rehydrating the GFR Matrigel inserts, the test substance was added to the wells. An equal number of Control Inserts (no GFR Matrigel coating) were prepared as control. 0.5 mL of T47-D cell suspension (2.5×10^4^ cells/mL) were added to the inside of the inserts. The chambers were incubated for 24 h at 37°C, 5% CO_2_ atmosphere. After incubation, the non-invading cells were removed from the upper surface of the membrane using cotton tipped swabs. Then the cells on the lower surface of the membrane were stained with Diff-Quick stain. The invading cells were observed and photographed under the microscope at 100×magnification. Cells were counted in the central field of triplicate membranes. The invasion index was calculated as the % invasion test cell/% invasion control cell.

### Tissue specimens

Wild-type and phosphorylated-moesin expression was investigated in 20 ductal invasive breast adenocarcinomas, stage pT1c. Five of these were ER^+^/N^+^, 5 were ER^+^/N^−^, 5 were ER^+^/N^+^ and 5 were ER^−^/N^−^. In addition, we also evaluated moesin and P-moesin in benign breast fibroadenomas (n = 5) as well as in normal breast tissue (n = 5). Samples were collected according to conventional histopathological diagnostic protocols and were fixed in 10% buffered formalin, embedded in paraffin, and stained with hematoxylin-eosin. Histological diagnosis and grading were performed according the *World Health Organization Classification of Tumors* (2002), and staging was determined according to the TNM system.

### Immunohistochemistry

Histological sections of 4 µm were mounted on silanized slides and allowed to dry for 1 h at room temperature (RT), followed by 1 h incubation in an oven at 60°C. Briefly, after deparaffination and rehydration, epitope retrieval was performed by immersing slides in DAKO Epitope Retrival Solution (0.01 M citrate buffer, pH 6.0) in a water bath at 98°C for 40 minutes followed by a 20 minutes cool-down period at RT. The working dilution for the anti-human moesin monoclonal antibody (clone 38; BD Biosciences) was 1∶50. P-moesin was evaluated with anti-P-moesin goat polyclonal antibody (Santa Cruz) at 1∶50 dilution. The sections were incubated with primary antibodies for 1 hour at RT. The DAKO EnVision detection system kit was employed to detect moesin, instead for P-moesin the DAKO LSAB+ was used. Moesin and P-moesin expression were scored as positive when there was cytoplasmic, membrane or both cytoplasmic and membrane immunostaining. Staining scores were established semi-quantitatively from the percentage of Moesin^+^ and P-moesin^+^ cells and the staining intensity. Tumors were graded as negative (<1% positive cells), focal (low intensity, with ≥1% to <30% positive cells), intense (moderate or high intensity with diffused positive cells). Staining patterns were independently adjudicated by two pathologists.

### Statistical analysis

All values are expressed as mean±SD. Statistical differences between mean values were determined by ANOVA, followed by the Fisher's protected least significance difference (PLSD).

The χ^2^ test, Fisher's exact test were used to evaluate the significance of the expression pattern of moesin and p-moesin and the clinical and pathological parameters with version 12 of the SPSS software package (SPSS Inc, IL, USA). A value of P<0.05 was considered significant.
